# Assessing the Potential Risks of Digital Therapeutics (DTX): The DTX Risk Assessment Canvas

**DOI:** 10.3390/jpm13101523

**Published:** 2023-10-23

**Authors:** Kerstin Denecke, Richard May, Elia Gabarron, Guillermo H. Lopez-Campos

**Affiliations:** 1 Department Engineering and Computer Science, Institute Patient-Centered Digital Health, Bern University of Applied Sciences, 3012 Bern, Switzerland; 2 Department of Automation and Computer Science, Harz University of Applied Sciences, 38855 Wernigerode, Germany; rmay@hs-harz.de; 3 Norwegian Centre for E-Health Research, University Hospital of North Norway, 9019 Tromsø, Norway; elia.gabarron@hiof.no; 4 Department of Education, ICT and Learning, Østfold University College, 1757 Halden, Norway; 5 Wellcome-Wolfson Institute for Experimental Medicine, Queen’s University Belfast, Belfast BT9 7BL, UK; g.lopezcampos@qub.ac.uk

**Keywords:** digital therapeutics, adverse event, patient safety, surveillance

## Abstract

Motivation: Digital therapeutics (DTX), i.e., health interventions that are provided through digital means, are increasingly available for use; in some countries, physicians can even prescribe selected DTX following a reimbursement by health insurances. This results in an increasing need for methodologies to consider and monitor DTX’s negative consequences, their risks to patient safety, and possible adverse events. However, it is completely unknown which aspects should be subject to surveillance given the missing experiences with the tools and their negative impacts. Objective: Our aim is to develop a tool—the DTX Risk Assessment Canvas—that enables researchers, developers, and practitioners to reflect on the negative consequences of DTX in a participatory process. Method: Taking the well-established business model canvas as a starting point, we identified relevant aspects to be considered in a risk assessment of a DTX. The aspects or building blocks of the canvas were constructed in a two-way process: first, we defined the aspects relevant for discussing and reflecting on how a DTX might bring negative consequences and risks for its users by considering ISO/TS 82304-2, the scientific literature, and by reviewing existing DTX and their listed adverse effects. The resulting aspects were grouped into thematic blocks and the canvas was created. Second, six experts in health informatics and mental health provided feedback and tested the understandability of the initial canvas by individually applying it to a DTX of their choice. Based on their feedback, the canvas was modified. Results: The DTX Risk Assessment Canvas is organized into 15 thematic blocks which are in turn grouped into three thematic groups considering the DTX itself, the users of the DTX, and the effects of the DTX. For each thematic block, questions have been formulated to guide the user of the canvas in reflecting on the single aspects. *Conclusions*: The DTX Risk Assessment Canvas is a tool to reflect the negative consequences and risks of a DTX by discussing different thematic blocks that together constitute a comprehensive interpretation of a DTX regarding possible risks. Applied during the DTX design and development phase, it can help in implementing countermeasures for mitigation or means for their monitoring.

## 1. Introduction

After having addressed quality requirements according to ISO/TS 82304-2 for evaluating the deployment of conversational agents in healthcare [1,2], in this paper, we dive deeper into the topic of assessing the risks associated with the use of digital therapeutics (DTX). DTX offer therapeutic interventions to patients delivered through high-quality software programs [3]. Similar to drugs or other treatments, they aim to cure, manage, or prevent disease. DTX can be used alone or in combination with other therapies, medical devices, or pharmaceuticals to improve patient care and health outcomes and are delivered as web-based disease prevention programs [4], conversational agents that deliver cognitive behavioral therapy [5,6], or in other ways.

The global DTX market size was estimated at USD 5.09 billion in 2022 and is expected to grow [7]. Applications related to diabetes and diabetes management dominated the global DTX market and held the largest revenue share of more than 28% in 2022. In 2022, North America held a commanding position in the digital therapeutics market, accounting for 40.7% of the market share. This can be attributed to the increasing implementation of healthcare spending reduction initiatives in the region, combined with a strong commitment to adopting a patient-centered approach to healthcare [7]. Some DTX can automatically adapt to the user’s needs and support active patient involvement in their care and disease self-management. For example, Woebot is a mental health chatbot that uses artificial intelligence (AI) and cognitive behavioral therapy techniques to provide mental health support to users [5]. EndeavorRx [8] is a DTX that aims to improve attention function in children aged 8–12 years with primarily inattentive or combined Attention Deficit Hyperactivity Disorder (ADHD) who have a demonstrated attention problem. It is the first FDA-approved ADHD treatment delivered through a video game.

Depending on their intended use, risk classification, and the regulations of the country in which a DTX is marketed, some DTX are classified as medical devices. For such DTX, some countries have already implemented a prescription and reimbursement process [9]. As of October 2023, the DTX Alliance lists nine countries in which DTX are available with a regulatory and reimbursement process in place (https://dtxalliance.org/ (accessed on 25 September 2023)). These include the United States, Japan, Singapore, South Korea, the UK, Australia, China, France, and Germany. For example, a regulatory and reimbursement pathway for DTX in the German market has been established [10] and the “fast-track” regulatory process for DTX was launched in 2019.

As they are used by individuals with or without the supervision of a healthcare practitioner, the quality of DTX is essential to avoid harming patients. However, we can recognize a lack of research on harm and the adverse effects of DTX and its current methodological imprecision [11]. A reason might be that it is still unclear which adverse reactions, responses, or risks can occur in the context of DTX. For example, one app that provides cognitive behavioral therapy for insomnia patients—which has been approved for reimbursement upon prescription in Germany—asks patients to keep a diary in the morning and evening using the app. However, no information is provided as part of the usage instructions and list of adverse effects that the blue light from the device might affect sleep and sleep quality. The effect has already been researched but obviously has not been reflected when developing the app [12]. This raises the question of whether developers of DTX are sufficiently reflecting on possible adverse reactions, responses, or risks (e.g., app–app interactions). Additionally, there is still no knowledge available about which negative consequences and risks can occur outside the controlled environment of clinical trials. Similar to drug–drug interactions, DTX can potentially interact with other digital health tools in a non-controlled environment [13]. Research and critical reflections on this are still missing.

To overcome this situation and ensure more patient safety, we believe it is necessary to carefully reflect on potential risks before DTX are released to market or tested on a large scale with patients. This paper therefore introduces a tool, a Risk Assessment Canvas for DTX. Its aim is to support a critical reflection on aspects that should be considered during the DTX development phase and for risk surveillance purposes when releasing DTX to the market, when prescribing a specific DTX to prepare for the broad range of negative effects and risks the use of DTX may cause, or for warning individuals before using a DTX. In previous work, we already recognized that the ISO Technical specification 82304-2 (ISO/TS 82304-2) Health software—Part 2: Health and wellness apps—Quality and reliability [2] provides relevant information to ensure high-quality conversational agents in healthcare [1]. In that paper, we linked quality requirements specified in the ISO/TS 82304-2 to global evaluation metrics defined for conversational agents in healthcare. Only a limited overlap was recognized, namely for the metrics related to ease of use, security, and accessibility. In this work, we will again use this technical specification in addition to other sources of information to identify relevant aspects for the risk assessment of DTX.

## 2. Materials and Methods

In this paper, we are suggesting a “DTX Risk Assessment Canvas” as a tool that supports a critical reflection on adverse reactions, events, and risks of a specific DTX. We are taking the Business Model Canvas developed by Osterwalder and Pigneur [14] as the basis for the development. It consists of nine “building blocks” that can be used to describe a business model. It is argued that a business model can be defined as a model that “describes the rationale for how an organization creates, delivers and captures value” [14] and that this definition can be captured by participants discussing all the “building blocks” of a business model. By discussing the different building blocks of a business model, such as key partners, channels, or revenue streams, it is possible to develop a comprehensive understanding of the way in which a company or organization is supposed to create, deliver, and capture value. In its original form, the business model canvas is used for a collaborative discussion. It has been adapted to other domains such as ethics [15].

Taking the business model canvas as a starting point, we defined building blocks to enable an interdisciplinary group of researchers, developers, practitioners, and potential users of a specific DTX to discuss and reflect on how this DTX might result in risks for users, the care process they are involved in, and the patient’s health. Similar to the business model canvas, we believe that by discussing the different aspects related to a DTX, such as risks, harms, or problematic use, it is possible to develop a comprehensive understanding of the adverse reactions, events, and risks of a DTX. This will help in developing countermeasures or establishing surveillance methodologies.

To achieve this, we collected different aspects that could contribute to adverse reactions, events, and risks of a DTX. The aspects or building blocks of the canvas were constructed in a two-way process: first, one author (KD) defined the building blocks. This was undertaken based on previous work related to quality of conversational agents in healthcare: KD considered the ISO/TS 82304-2, which was first published in August 2020 [2]. It is based upon guidelines and requirements for apps. Its purpose is to ensure that health and wellness apps are safe, reliable, and effective. The technical specification is intended for use by app manufacturers as well as app assessment organizations in order to communicate the quality and reliability of a health app. It groups quality aspects into 5 categories: product information, healthy and safe, easy to use, secure data, and robust build. It has already been considered for collecting aspects for an evaluation framework for conversational agents in healthcare. Therefore, KD assessed the quality requirements listed in this specification and selected aspects that might be of interest for assessing aspects related to the risks associated with a DTX. Additionally, she studied the relevant literature and the product information of the DTX listed in the DTX repository of the German Authorities for regulating drugs and medical devices (https://diga.bfarm.de/de/verzeichnis (accessed on 25 September 2023)). At the time of reviewing these, 54 DTX were listed in the repository. Relevant aspects were collected and aggregated into groups that formed at the end the thematic blocks of the canvas.

In a second step, 6 experts in health informatics and mental health provided feedback and tested the understandability of the canvas by individually applying it to a DTX of their choice. None of the experts were introduced to the canvas before the test. They were provided with a brief introduction to the DTX Risk Assessment Canvas and its objectives, including its expected use. They were asked to consider a concrete DTX and reflect on the aspects listed in the canvas. Additionally, they were asked to provide feedback on the process of applying the canvas. The experts’ input was used to adapt the canvas and the guiding questions. The resulting canvas will be described in Section 3.

## 3. Results

### 3.1. DTX Risk Assessment Canvas

**Figure 1:**
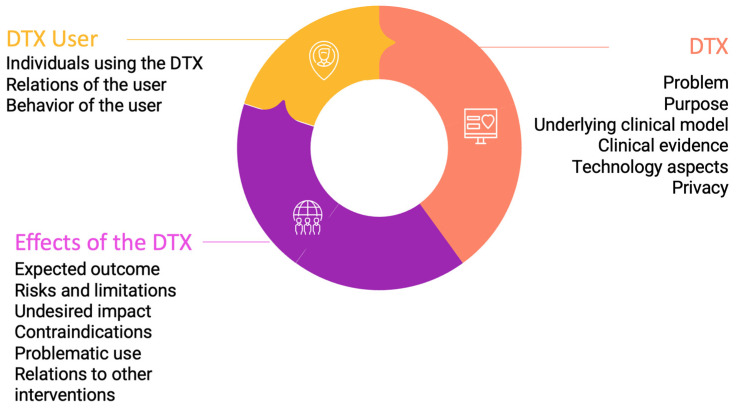
Overview of the three thematic groups and 15 thematic blocks of the DTX Risk Assessment Canvas.

**Figure A1:**
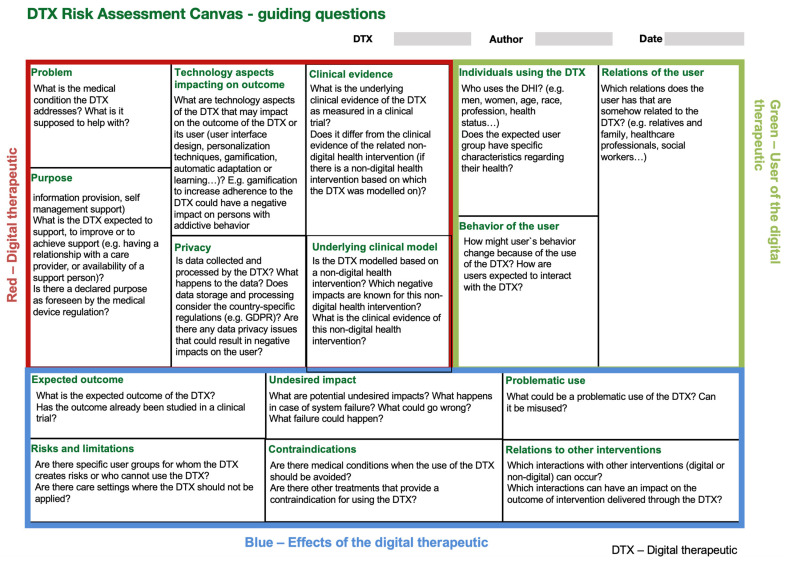
DTX Risk Assessment Canvas and Guiding Questions.

The DTX Risk Assessment Canvas is organized into 15 thematic blocks. They are grouped together into three thematic groups (see Figure 1): DTX (Section 3.1.1), users of the DTX (Section 3.1.2), and effects of the DTX (Section 3.1.3). For each block, guiding questions have been formulated to encourage researchers, developers, and practitioners to reflect on the individual thematic blocks (Table 1 and Figure A1).

#### 3.1.1. Thematic Blocks Related to the DTX

The group DTX considers aspects related to the digital solution that are described by six thematic blocks: problem, purpose, clinical model, clinical evidence, technology aspects, and privacy [16]. First, the problem the DTX addresses should be specified when reflecting the possible risks of a DTX. Guiding questions include: which medical condition is addressed or what is the DTX supposed to support? This aspect is of relevance since there may be risks associated with the medical condition the DTX is targeting. For example, Yang and Li studied the “dark side” of gamification for healthcare management support and found “that both privacy invasion and social overload are positively associated with users’ gamification exhaustion” [17].

The second aspect to be reflected related to the DTX is the purpose. We define purpose as the aim or goal of the DTX. The purpose of a DTX has to be described, in particular when assigning a DTX to one of the medical device classes defined by the medical device regulations [18]. However, since the canvas is also relevant for DTX that are not considered medical devices, we consider specifying the purpose as relevant for all DTX. The purpose may impact the care process where a DTX will be integrated and where risks could be associated with.

Two other thematic blocks related to the solution consider the underlying clinical model and the clinical evidence. Sometimes an existing clinical model is digitized in a DTX, so the risks or negative impacts of this clinical model could also be relevant to the digital version. For example, the chatbot Woebot integrates the clinical model of cognitive behavioral therapy [5,19]. Possible risks associated with this type of therapy might have already been studied for its non-digital delivery.

A non-digital health intervention is assessed regarding efficacy and safety in clinical trials (phase III). This is summarized as clinical evidence. A similar concept has been defined for medical devices: According to the International Medical Device Regulators Forum, clinical evidence is “the clinical data and the clinical evaluation report pertaining to a medical device.” [20]. Given the missing knowledge of safety aspects related to DTX, the DTX Risk Assessment Canvas asks to reflect on the non-digital health intervention that may underly a DTX (i.e., the clinical model) and its effects and evidence. Our canvas therefore asks for reflecting on the clinical evidence of the DTX.

Another important aspect regarding the DTX itself is technology aspects that may have an impact on the outcome of the DTX or its users. Within this thematic block, it is important to reflect on aspects such as personalization techniques, the realization of interactions between the user and the DTX (e.g., an empathetic chatbot who claims to be a best friend could have an impact on social contacts in the real world), or technology aspects such as blue light transmitted by the screen of the smartphone or the PC screen.

The sixth thematic block related to the DTX itself considers privacy which is strongly related to data processing and storage as well as associated security mechanisms [16]. Data misuse or reuse for different purposes may have negative impacts on the users of a DTX [21], even in the context of their safety.

#### 3.1.2. Thematic Blocks Related to the User of a DTX

A second group of aspects in the canvas addresses three aspects related to the user of a DTX: user, relations of the user, behavior of the user. The idea behind the user block is to help in identifying aspects related to the health or sociodemographic aspects of the user that may be problematic when using a DTX. It aims to capture details such as cultural aspects of the expected user group.

In certain situations, the use of a DTX may also impact the relationships with other individuals associated with the DTX user such as relatives or friends. Related to this, risks or undesired impacts can occur (which are then reflected in the third thematic block under “undesired impact”). Therefore, the second thematic block in this group concerns the relations of the user.

The third thematic block asks to reflect on the behavior of the user. Here we are asking to think about potential changes in the user’s behavior because of the use of a DTX and for the expected interaction with the DTX. As exemplified, the above mentioned therapeutic video game EndeavorRx [8] for children with primarily inattentive or combined Attention Deficit Hyperactivity Disorder led to an increased aggressivity in some kids. It could have been reflected in advance, i.e., before releasing the DTX, that playing videogames can lead to negative behavior changes in kids. Even though this could not be changed, it could be indicated as a possible effect to be surveilled as part of the surveillance process of this DTX.

#### 3.1.3. Thematic Blocks Related to the Effects of a DTX

The third group deals with the effects of a DTX and consists of six thematic blocks: Expected outcome, risks and limitations, contraindications, undesired impact, problematic use, and relations to other interventions. First, the expected outcome should be reflected. What is the DTX expected to support, to improve, or to achieve? We are also asking whether the outcome has already been studied in a clinical trial.

Second, risks and limitations should be collected. A DTX could create risks for a specific user group or specific users might be prevented from using a DTX. For example, people with visual impairment may have problems with interacting with a text-based conversational agent (or when they use it, the risk for wrong usage behavior could increase). Potential harms caused by gamification elements should be considered here when gamification is a technology aspect of the DTX. There might be also care settings in which a DTX should not be applied. These aspects are asked to be reflected.

Third, contraindications are collected. In this item, contraindications of the underlying clinical model should be reflected. Although it might be still unknown whether the DTX will have the same contraindications as the underlying clinical model, a critical reflection might already be useful to create an awareness of potential risks.

Fourth, reflections on problematic use of the DTX are requested by the canvas. Can the DTX be misused by users or used in a way that leads to health issues or other negative impacts? These considerations are important to develop mitigation strategies for problematic use or—when the DTX is supposed to be integrated in a care process—to create sensitivity to the potential risk of misuse by the care provider.

The fifth thematic block related to the effects of the DTX addresses interactions with other interventions (digital or non-digital, regulated or unregulated interventions). For example, the exposure to other digital contents might affect the use or outcome of a DTX [22]. An example of this type of interaction would be interactions of the user with social media and their influence on the outcomes of a DTX addressing the eating disorder of the user [23].

Sixth, it should be reflected which inferences of potential undesired impacts the DTX may have. For this reflection, the information on the user and the DTX as collected by the thematic blocks in the other two groups as well as of the other five thematic blocks of this group are relevant to be considered. This thematic block is probably the core of the canvas. As exemplified, a DTX can impact the relationship with the healthcare provider. This clearly depends on the integration into the care flow. When a user uses a DTX accompanying the standard therapy without letting the treating healthcare provider know, adverse events cannot be recognized; trust in the healthcare provider could be impacted, etc. The technological aspects collected in the first block can lead to undesired impacts. Besides this, we are asking to think about situations of system failure, specifically which failures could occur in a real-world setting.

#### 3.1.4. Expected Use of the DTX Risk Assessment Canvas

**Figure A2:**
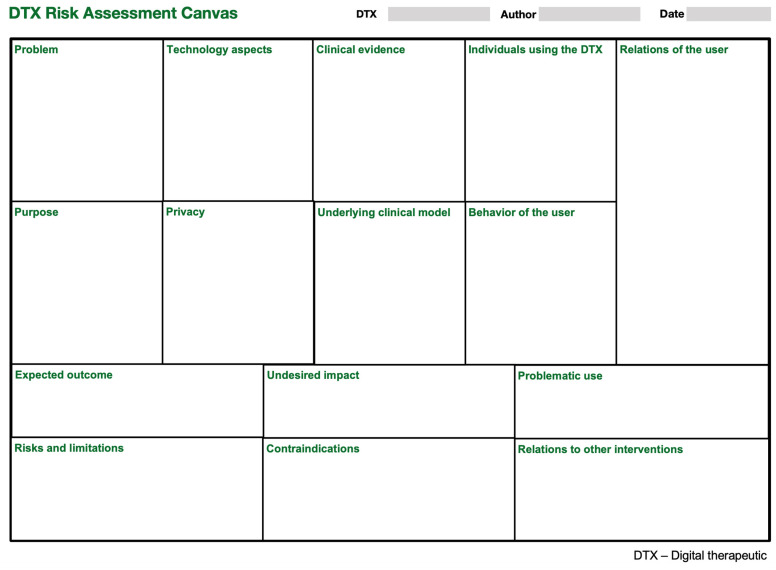
DTX Risk Assessment Canvas Template.

We expect the use of the DTX Risk Assessment Canvas to be in a participatory discussion process among developers of the DTX, healthcare professionals and other groups of persons that might be involved in the process the DTX is supposed to be used in, and researchers. The participants discuss the aspects defined by the 15 thematic blocks. Further, potential users of the DTX under consideration could be involved in this reflection process. Specifically, the canvas is used by the group to reflect on risks associated with the DTX. First, the group will collect and aggregate the information on the DTX (thematic group 1) and its users (thematic group 2). Once this has been undertaken, the third thematic group is used to assess potential risks. Another option is that the participants are reflecting on the 15 thematic blocks in an individual manner and meet afterwards to discuss and aggregate their individually collected thoughts in the group discussion. To support this process, we are providing a sheet with the 15 thematic blocks to be filled (Figure A2) and a sheet with the guiding questions per block (Figure A1).

When applied during the conceptual or development phase of a DTX, the collected possible risks and adverse events can be considered in order to implement possible mitigation strategies. For harms and risks where no mitigation strategy can be found, a clear announcement in terms of possible contraindications or risks associated with DTX use should be provided to all users of a DTX. Also, surveillance measures can be put in place to at least monitor the risks.

To facilitate working with the canvas in multidisciplinary teams, we created a glossary of terms with definitions of the most important terms (Table A1). Since the participants in the risk assessment process can originate from different fields, it has to be ensured that a common terminology is used.

## 4. Discussion

### 4.1. Relevance to Prior Work

With regulations released in recent years (e.g., the EU Medical Device Regulation), there are DTX that are classified as medical devices and are now subject to similar development and approval processes as drugs and medical devices. They are tested on selected volunteers and patients prior to market launch to verify their efficacy and safety. Their effectiveness of use must be proven through systematic clinical trials [24] that assess the outcome in controlled settings to reduce bias. In fact, the most often chosen study design for DTX is randomized controlled trials (RCT) [25]. When reading through the instructions of use for the DTX currently approved for prescription in Germany [9], we can recognize that the surveillance of a DTX relies upon active reporting from the users and eventually healthcare providers. All apps listed in the German repository confirm that no adverse effects were recognized in the trial or testing phase (September 2023). Even obvious contraindications such as the one described in the introduction (blue light having an impact on sleep quality [26]) remain undescribed in the usage instructions.

In pharmacological treatment, assessment of harm takes place in all phases of the clinical trials, from the early preclinical and basic science phases of the development (Phase I) to the postmarketing stage (Phase IV). In contrast, DTX are typically studied in single-phased RCTs aimed at evaluating their efficacy or observation studies focused on assessing their clinical efficacy and comparison with current treatments, omitting in-depth harm assessments during treatment development [27]. Beyond this, the need for conducting a clinical trial and assessing adverse effects and efficacy does only apply for DTX that are classified as medical devices. Ensuring user safety would be necessary for any DTX available to individuals.

For drugs, a monitoring process called pharmacovigilance has become mandatory in order to collect risks and adverse effects after the market release of a drug. A similar approach was suggested with upcoming Artificial Intelligence (AI) applications in healthcare. The concept of “Algorithmovigilance” introduced by P. Embi in 2021 is an approach to evaluate systematically AI-enabled health interventions [28]. It focuses basically on the AI algorithms, their development, and related biases. Recently, we defined the field of digitalovigilance as a research field for collecting, detecting, assessing, monitoring, and preventing adverse effects caused by DTX [13]. However, only risks or events that are known can be surveilled in such a process.

The DTX Risk Assessment Canvas therefore aims to support analyzing the complexities and challenges related to DTX. DTX often involve a combination of technology, healthcare processes, patient engagement, and data management. The canvas provides a structured framework to consider and reflect on these multifaceted aspects. It also helps in assessing the impacts of a DTX in a landscape of diverse stakeholders. We intentionally did not include regulatory and ethical considerations related to DTX in order to focus on the technical-related risks and adverse events. There are other tools and frameworks available to address these aspects. For example, the Digital Therapeutics Alliance formulated a DTX Industry Code of Ethics [29]. The Ethics Canvas provided by the ADAPT Centre and Trinity College Dublin is a tool that supports reflection on the ethical aspects of solutions (not necessarily digital health solutions) [30].

A common process for assessing the value of a health technology is health technology assessment (HTA): “*HTA is a multidisciplinary process that uses explicit methods to determine the value of a health technology at different points in its lifecycle. The purpose is to inform decision-making in order to promote an equitable, efficient, and high-quality health system*” [31]. Traditional HTA does not cover all factors relevant to digital tools, such as accessibility and data security and protection [32]. To address this issue, Haverinen et al. adapted the HTA framework for realizing the HTA process for digital healthcare services. The framework was named Digi-HTA [33]. It contains aspects that are related to ours in the DTX Risk Assessment Canvas; for example, it asks for safety issues related to robotics and AI. As in the traditional HTA process, the effectiveness is of importance as well. In our framework, we included clinical evidence. However, the Digi-HTA includes aspects such as usability and interoperability as well as costs, which are not part of our canvas. The overlapping aspects show the relevance of aspects such as data security or technology aspects on patient safety. In addition to the Digi-HTA, other resources have been developed for digital health technology assessment across different regions in the world: the Digital Technology Assessment Criteria for Health and Social Care (DTAC) in the UK, the Digital Health Assessment Framework (DHAF) in the US, NorDEC in Nordic countries in Europe, and the overarching ORCHA. As was previously discussed in the context of Digi-HTA, these HTA solutions cover areas that are not covered in our canvas. Table 2 presents the similarities and differences between these different digital health technology assessment tools and our DTX Risk Assessment Canvas. The overlapping domains across all these tools are privacy, clinical evidence, and functionality and purpose, whereas other relevant domains in regulatory DHTA such as usability or interoperability are not covered in the DTX Risk Assessment Canvas. Many of these other approaches represent a comprehensive process to be realized when the implementation of the digital solution has been completed, while our DTX Risk Assessment Canvas is supposed to be used in the development and design phase to address relevant aspects already in the development phase. Beyond this, our canvas should also be used for DTX that are not considered medical devices to ensure user safety. Digi-HTA and HTA processes are normally only applied to medical devices since the assessment process is very comprehensive and time-consuming.

There are also technical specifications for health and wellness apps addressing aspects around quality and reliability developed by the ISO committee. We considered these specifications in the definition of the thematic blocks and guiding questions. We retrieved some input for our DTX Risk Assessment Canvas. For example, the ISO/TS 82304-2 contains the subcategories “health risks” and “health benefit” as well as “privacy” and “security”. However, the technical specification is intended to be used by developers, manufacturers, and regulatory bodies to assess and improve the performance of health software applications. It offers guidance on various aspects of app development, such as user interface design, data security, interoperability, and usability. It is a comprehensive guideline covering multiple aspects. In contrast, our DTX Risk Assessment Canvas is more focused on the negative consequences and adverse events of DTX and also considers the processes a DTX is supposed to be used in.

### 4.2. Strengths and Limitations

This is, to our knowledge, the first attempt at supporting a critical reflection on possible adverse events for DTX. In this way, we offer a guidance for reflection that could hopefully support the development of countermeasures for potential serious adverse events, or at least a warning towards the users. The DTX Risk Assessment Canvas is based on the literature, the ISO/TS 82304-2, and on existing evidence in terms of DTX and their contraindication and adverse events, as well as expert knowledge.

So far, the canvas is a proof of concept prototype that was tested by a limited number of users. It is clear that this cannot ensure the understandability and completeness of the canvas. A more comprehensive evaluation is needed to ensure understandability. However, the canvas is intended to provide a basis for thinking about and discussing possible adverse reactions. This does not necessarily require that for all possible aspects questions are contained in the canvas.

## 5. Conclusions

In an increasingly digitalized world, the role of digitalovigilance for the detection and study of the potential interactions between the different digital health components will be key to the safe use of DTX and their integration in care processes.

Future research will have to study effects on patient safety and outcomes when different DTX are combined and evaluated together, similar to the way combined drug therapies are currently used. Although a complete assessment of the adverse effects of DTX and their interactions is impossible, it is important to recognize and consider them. The DTX Risk Assessment Canvas provides a tool to reflect the possible risks and negative consequences of a DTX by discussing different thematic blocks that together constitute a comprehensive interpretation of a DTX regarding aspects relevant to be considered within surveillance of a DTX. It is expected that it will raise awareness for the need for a systematic assessment of risks associated with the use of DTX and for the discipline “digitalovigilance”, ensuring continuous monitoring of such negative impacts going beyond the regulatory minimum. Consequently, our next steps are to first validate the usage of the canvas in workshops and to derive from this validation phase a workshop concept that can be used by persons developing a DTX. Additionally, the validation phase would result in an improved knowledge of the possible risks of a DTX, which helps in developing monitoring measures. We expect the best time to use the canvas is during the design stage since many options in terms of development and realization are still open, which will allow the implementation of countermeasures to the potential risks right from the beginning. However, it could also be applied in later development stages or during use time to tailor surveillance methods. This still has to be studied.

## Figures and Tables

**Table 1:** The DTX Risk Assessment Canvas including the guiding questions.

	Guiding Questions
DTX	
Problem	What is the medical condition the DTX addresses? What is it supposed to help with?
Purpose	What is the intended purpose of the DTX? (e.g., coaching, diagnosing, information provision, self-management support) What is the DTX expected to support, to improve, or to achieve support (e.g., having a relationship with a care provider or availability of a support person)? Is there a declared purpose as foreseen by the medical device regulation?
Underlying clinical model	Is the DTX modelled based on a non-digital health intervention (e.g., cognitive behavior therapy)? Which one? Which negative impacts are known for this non-digital health intervention? What is the clinical evidence of this non-digital health intervention (i.e., efficacy and safety results measured by a clinical trial)?
Clinical evidence	What is the underlying clinical evidence of the DTX as measured in a clinical trial? Does it differ from the clinical evidence of the related non-digital health intervention (if there is a non-digital health intervention based on which the DTX was modeled)?
Technology aspects that may impact outcome or the individual	What are technology aspects of the DTX that may impact the outcome of the DTX or its user (user interface design, personalization techniques, gamification, automatic adaptation, or learning…)? E.g., using gamification to increase adherence to the DTX could have a negative impact on persons with addictive behavior.
Privacy	Are data collected and processed by the DTX? What happens to the data? Does data storage and processing consider country-specific regulations (e.g., GDPR)? Are there any data privacy issues that could result in negative impacts on the user?
User of the DTX	
Individuals using the DTX	Who uses the DTX? (e.g., men, women, age, race, profession, health status…) Does the expected user group have specific characteristics regarding their health?
Relations of the user	What relations does the user have that are somehow related to the DTX? (e.g., relatives and family, healthcare professionals, social workers…).
Behavior of the user	How might the user’s behavior change because of the use of the DTX? How are users expected to interact with the DTX?
Effects of DTX	
Expected outcome	What is the expected outcome of the DTX? Has the outcome already been studied in a clinical trial?
Risks and limitations	Are there specific user groups for whom the DTX creates risks or who cannot use the DTX? Are there care settings in which the DTX should not be applied?
Contraindications	Are there medical conditions for which the use of the DTX should be avoided? Are there other treatments that provide a contraindication for using the DTX?
Undesired impact	What are the potential undesired impacts of the DTX? What happens in case of system failure? Which technology aspects might impact the outcome of the DTX (e.g., blue light can cause sleep problems)? What could go wrong? What failure could happen? How may relations of the user change through the use of the DTX? (e.g., patient–doctor relationship, family).
Problematic use	What could be a problematic use of the DTX? Can it be misused?
Relations to other interventions	What interactions with other interventions (digital or non-digital) can occur? What interactions can have an impact on the outcome of the intervention delivered through the DTX?

**Table 2:** This table presents the similarities and differences between different digital health technology assessment (DHTA) tools and the proposed DTX Risk Assessment Canvas.

Digital Health Technology Assessment Tool	No. Domains	Domains Details	Overlapping Concepts with DTX Risk Assessment Canvas
DTX Risk Assessment Canvas	3	DTX description User of the DTX Effects of the DTX	
The Digital Technology Assessment Criteria for Health and Social Care (DTAC) (UK) ^1^	5	Clinical Safety Data Protection Technical security Interoperability criteria Value proposition (not assessed)	Privacy, clinical evidence, functionality and purpose, and intended users
ORCHA Baseline review (OBR) ^2^	3	Clinical or professional assurance Data and privacy Usability and accessibility	Privacy, clinical evidence, and functionality and purpose
Digi-HTA [33]	11	Company information Product information Technical stability Usability and accessibility Interoperability Cost Effectiveness Clinical safety Data security and protection Artificial intelligence Robotics	Privacy, clinical evidence, and functionality and purpose
Digital Health Assessment Framework (DHAF) (US) ^3^	4	Data and Privacy Clinical assurance and safety Usability and accessibility Technical security and stability	Privacy, clinical evidence, and functionality and purpose
NorDEC (Nordic countries Europe) ^4^	5	Data and Privacy Professional Assurance and clinical safety Usability and accessibility Security and technical stability Interoperability	Privacy, clinical evidence, and functionality and purpose

^1^ https://transform.england.nhs.uk/key-tools-and-info/digital-technology-assessment-criteria-dtac/ (accessed on 17 September 2023), ^2^ https://orchahealth.com (accessed on 17 September 2023), ^3^ https://dhealthframework.org (accessed on 17 September 2023), ^4^ https://norddec.org (accessed on 17 September 2023).

## Data Availability

Not applicable.

## Appendix A

**Table A1:** Definitions of Terms Attached to the Canvas.

Term	Definition
Adverse event	An adverse event is an unexpected and undesirable occurrence or outcome that happens during or after a medical treatment, intervention, or the use of a DTX. Adverse events can range from mild side effects to severe complications and may include reactions to medications, medical procedures, medical device malfunctions, or incidents related to healthcare delivery. The identification, reporting, and analysis of adverse events are crucial in healthcare to monitor and improve the safety and effectiveness of treatments and interventions.
Clinical evidence	Clinical evidence refers to the information and data obtained from clinical research studies and trials that provide insights into the effectiveness, safety, and potential benefits or risks of medical treatments, interventions, therapies, or procedures. This evidence is gathered through systematic scientific research involving human participants under controlled conditions and is a fundamental component of evidence-based medicine.
Clinical model	Therapeutic approach underlying a non-digital health intervention.
Contraindication	A contraindication or counter-indication is a circumstance that prohibits the use of a diagnostic or therapeutic procedure in the case of a given indication or only permits it after strict consideration of the risks involved.
Digital Therapeutic (DTX)	DTX provide patients with evidence-based therapeutic interventions. They are delivered through high-quality software programs.
Gamification	Gamification is the practice of incorporating game-like elements, such as points, challenges, and rewards, into DTX to engage and motivate individuals, encouraging desired behaviors and achieving specific goals. It aims to make tasks or interactions with a DTX more enjoyable and interactive, often enhancing engagement and adherence.
Harm	Harm refers to any adverse effect or negative outcome experienced by individuals as a result of using DTX. This can include physical harm, such as health complications arising from the use of a medical app, as well as privacy breaches, emotional distress, or misinformation that may result from the interaction with DTX.
Impact	Impact in the context of DTX refers to the measurable and often intended outcomes or effects resulting from the implementation and use of DTX. These impacts can be categorized as follows: Expected Impact: These are the anticipated and planned positive outcomes that DTX aim to achieve. Expected impacts may include improved patient outcomes, enhanced access to healthcare services, increased efficiency in healthcare delivery, cost savings, and better management of health conditions. These effects are typically part of the intervention’s intended goals and objectives. Undesired impact: These are unanticipated consequences, whether positive or negative, that arise from the use of DTX. Undesired impacts can include unanticipated benefits or risks that were not initially foreseen during the development and implementation of the intervention. These effects may emerge as users engage with the technology, and they may require adjustments or further evaluation to address.
Problematic use	Problematic use of a DTX refers to when an individual excessively relies on or becomes overly preoccupied with a DTX or applies it for other purposes then foreseen, leading to negative impacts on their well-being or health outcomes. This can include spending too much time using the tool, prioritizing it over professional advice, experiencing negative emotions related to its use, and even neglecting other aspects of their life, potentially harming their health.
Privacy	Privacy refers to the protection of individuals’ personal health information and data collected, processed, or shared through DTX. It involves ensuring that sensitive health-related data are kept confidential and secure.
Risk	Risk refers to the potential for adverse outcomes or harm associated with the use or deployment of DTX. These risks can include issues related to data security and privacy, inaccurate health information, user dependence, and negative health consequences resulting from the intervention.
